# Determination of the Structural Integrity and Stability of Polysialic Acid during Alkaline and Thermal Treatment

**DOI:** 10.3390/molecules25010165

**Published:** 2019-12-31

**Authors:** Bastian Bartling, Johanna S. Rehfeld, Daniel Boßmann, Ingo de Vries, Jörg Fohrer, Frank Lammers, Thomas Scheper, Sascha Beutel

**Affiliations:** 1 Institute of Technical Chemistry, Leibniz University Hannover, 30167 Hannover, Germany; bartling@iftc.uni-hannover.de (B.B.); rehfeld@iftc.uni-hannover.de (J.S.R.); bossmann@iftc.uni-hannover.de (D.B.); devries@iftc.uni-hannover.de (I.d.V.); scheper@iftc.uni-hannover.de (T.S.); 2 Institute of Organic Chemistry, Leibniz University Hannover, 30167 Hannover, Germany; joerg.fohrer@oci.uni-hannover.de; 3 Sanofi-Aventis Deutschland GmbH, Industriepark Hoechst, 65929 Frankfurt am Main, Germany; frank.lammers@sanofi.de

**Keywords:** polysialic acid, endotoxin removal, structure integrity, hydrolysis, NMR spectroscopy

## Abstract

Polysialic acid (polySia) is a linear homopolymer of varying chain lengths that exists mostly on the outer cell membrane surface of certain bacteria, such as *Escherichia coli* (*E. coli*) K1. PolySia, with an average degree of polymerization of 20 (polySia avDP20), possesses material properties that can be used for therapeutic applications to treat inflammatory neurodegenerative diseases. The fermentation of *E. coli* K1 enables the large-scale production of endogenous long-chain polySia (DP ≈ 130) (LC polySia), from which polySia avDP20 can be manufactured using thermal hydrolysis. To ensure adequate biopharmaceutical quality of the product, the removal of byproducts and contaminants, such as endotoxins, is essential. Recent studies have revealed that the long-term incubation in alkaline sodium hydroxide (NaOH) solutions reduces the endotoxin content down to 3 EU (endotoxin units) per mg, which is in the range of pharmaceutical applications. In this study, we analyzed interferences in the intramolecular structure of polySia caused by harsh NaOH treatment or thermal hydrolysis. Nuclear magnetic resonance (NMR) spectroscopy revealed that neither the incubation in an alkaline solution nor the thermal hydrolysis induced any chemical modification. In addition, HPLC analysis with a preceding 1,2-diamino-4,5-methylenedioxybenzene (DMB) derivatization demonstrated that the alkaline treatment did not induce any hydrolytic effects to reduce the maximum polymer length and that the controlled thermal hydrolysis reduced the maximum chain length effectively, while cost-effective incubation in alkaline solutions had no adverse effects on LC polySia. Therefore, both methods guarantee the production of high-purity, low-molecular-weight polySia without alterations in the structure, which is a prerequisite for the submission of a marketing authorization application as a medicinal product. However, a specific synthesis of low-molecular-weight polySia with defined chain lengths is only possible to a limited extent.

## 1. Introduction

**Figure 1:**
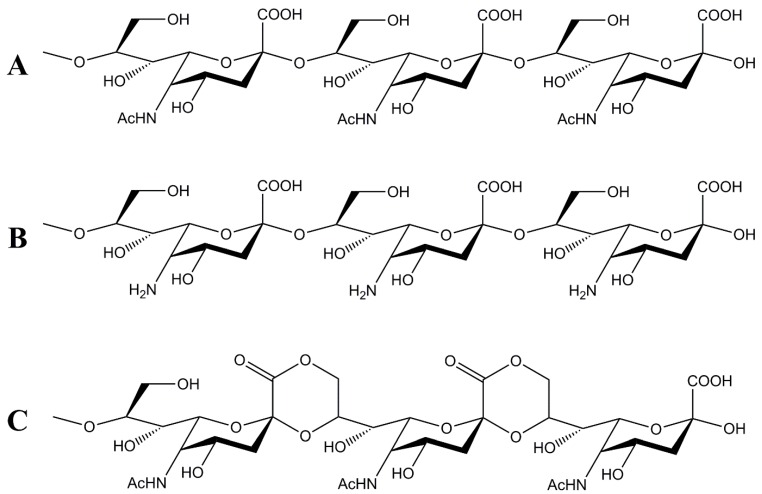
Chemical structure of α-(2,8)-linked derivates of polysialic acid: (**A**) structure of the homopolymer of α-(2,8)-linked 5-N-acetylneuraminic acid (polySia), (**B**) homopolymer of α-(2,8)-linked neuraminic acid, and (**C**) the lactonized structure of α-(2,8)-linked polySia.

Polysialic acid (polySia) is a linear homopolymer consisting of sialic acid monomers. Sialic acid is a derivative of neuraminic acid, which normally contains an acetylated amino function on the C_5_ atom, leading to N-acetylneuraminic acid (Neu5Ac) [1,2]. PolySia chains of varying lengths contain large numbers of α-(2,8)- or α-(2,9)-glycosidically-bound Neu5Ac residues. Some prokaryotes like *Neisseria meningitis (N. meningitis)* or *Escherichia* (*E. coli*) K1 synthesize a human-identical α-(2,8)-linked form of polySia (Figure 1A) as a capsular polysaccharide and virulence factor.

The polysaccharide masks the underlying cellular surface molecules of the pathogens [3]. The bacteria are thus protected from the host’s defense mechanisms and are even able to cross the blood–brain barrier [4]. In vertebrates, α-(2,8)-linked polySia is found as a terminal posttranslational modification on the surface of glycoproteins, e.g., the neural cell adhesion molecule (NCAM) [5,6,7]. Thereby, the molecule acts as a chemical messenger modulating cell–cell interaction and cell–molecule recognition processes [8,9]. Additionally, polySia has been ascribed important roles in neural development and cell differentiation [10]. Interestingly, low-molecular-weight polySia with an average degree of polymerization of 20 (avDP20) has an anti-inflammatory effect on human THP1 macrophages, and therefore suppresses the complement system-mediated autoimmune response [6,11]. Due to these biochemical properties, polySia avDP20 in particular holds potential for the development of biomedical applications to treat neurodegenerative diseases [12].

In recent decades, great efforts have been made to improve the production of polySia using *E. coli* [13,14]. Recent studies have described a complete manufacturing process that also enables the large-scale production of LC polySia (long-chain polySia with a DP ≈ 130) [15,16]. These novel processes are based on disposable systems to facilitate production according to the regulatory requirements of good manufacturing practice (GMP) [17].

GMP-compliant manufacturing processes are a key prerequisite to obtaining pharmaceutical products of a specified and reproducible quality. The level of process- and product-related impurities in these processes need to be controlled within a proven acceptable range. The endotoxin level is one of the most critical process parameters related to impurities, and the reduction of endotoxins to a specified maximum level is consequently essential for pharmaceutical applications.

In particular, reducing the high endotoxin content in the fermentation broth to an acceptable level poses a particular challenge for downstream processing in the production of high-purity polySia. Endotoxins are negatively charged lipopolysaccharides (LPS) that can cause inflammatory reactions, even in very low concentrations (1 ng per kg body weight and hour) [18], and thus need to be controlled in order to enable the therapeutic application of polySia [19]. Since polySia also has a very similar anionic character and molecular weight to endotoxins, most purification techniques are not efficient at the removal of endotoxins from polySia.

A cost-effective method to efficiently remove endotoxins has been described for the production of poly(3-hydroxybutyrate) from *E. coli*. During downstream processing, the product was incubated in a sodium hydroxide (NaOH) solution [20]. Since no product degradation was described for this method, de Vries et al. adopted this method for the production process of LC polySia. The final exposure of the product was determined with 3 EU (endotoxin units) per mg. However, long-term incubation in alkaline NaOH solution is suspected to cause deacetylation of the amino function, resulting in the formation of α-(2,8)-linked neuraminic acid (Figure 1B) [7,21], and may change the polymer chain length due to possible alkaline hydrolysis. This entails the risk of generating product-related impurities that could negatively impact critical quality attributes with a resulting loss of the desired biological activity.

A further challenge remains in the production of sufficient quantities of low-molecular-weight polySia with defined chain lengths for biomedical applications as only LC polySia can be produced on an industrial scale [22,23]. Production processes for polysaccharides from natural sources suffer from difficulties in the extraction and purification of the product [24]. A further disadvantage lies in the batch-dependent variations in size and the related chain length of the polysaccharides [25]. A promising approach for the synthesis of polysaccharides with defined chain lengths is the cleavage of long-chain material through controlled enzymatic or thermal hydrolysis [26,27]. The acid-catalyzed hydrolysis of LC polySia also enables the degradation of polysaccharides [28]. However, the acidic conditions also favor the internal δ-lactonization between the carboxyl group and the hydroxy group on the C_9_ atom (Figure 1C). These changes in the monomer structure may lead to the loss of antigenicity and biological activity for the molecule [29,30,31]. Interestingly, LC polySia decomposes at room temperature within a few days. This temperature sensitivity can be used efficiently for polySia avDP20 production, as an increase in temperature accelerates the process. The chain-length distribution of the degradants can be influenced by varying the hydrolysis time.

Appropriate stability investigations are typically used to understand the shelf-life and/or structural changes of a product over time or under process conditions. In combination with impurity clearance studies, both enhance the understanding of processes and products and are one of the key requirements for the submission of a marketing authorization application.

In this study, we were able to show that the described method neither induced structural changes in the target product nor resulted in the cleavage of polysialic acid chains. This was demonstrated via intramolecular structural analyses, performed using ^1^H- and ^13^C-nuclear magnetic resonance (NMR) spectroscopy. In addition, the maximum chain length was analyzed using common 1,2-diamino-4,5-methylenedioxybenzene (DMB)-HPLC.

## 2. Results

### 2.1. Endotoxin Removal of Biotechnologically Produced PolySia

Manufactured LC polySia [15] with a high endotoxin burden (>3000 EU per mg) was incubated in a sodium hydroxide solution (pH > 13) and recovered via an anion exchange (AEX) membrane adsorber (SartobindQ 75 mL) to fully remove endotoxins. A second parallel experiment used the same initial material for the application of specific endotoxin-removal columns (EndoTrap HD). Incubation of polySia in alkaline NaOH solution and its subsequent recovery via SartobindQ 75 mL reduced the endotoxin burden to less than 5 EU per mg. Using EndoTrap HD columns as a control under physiological-like conditions (pH ≈ 7), the endotoxin content was reduced to less than 2 EU per mg. However, approximately 13% of the product was lost according to the conducted thiobarbituric acid (TBA)-assay [32,33]. In contrast, the incubation of polySia in NaOH resulted in no significant reduction in yield. Thus, both procedures represent simple and efficient ways to remove the endotoxin burden of produced polySia to achieve a product of pharmaceutical grade. Furthermore, the results are comparable to earlier studies [16].

### 2.2. Analysis of Maximum Chain Length of NaOH-Treated and Untreated PolySia

However, it is also important to ascertain that strong alkaline conditions do not alter the molecular structure of polySia. To this end, this study also focused on demonstrating that biotechnologically produced polySia was not negatively affected by the harsh alkaline solutions used for the removal of endotoxins. After purification, the material was dialyzed against deionized water and lyophilized. The samples were then DMB-derivatized and the maximum chain length was subsequently analyzed via AEX HPLC in combination with fluorescence detection [34]. In all cases, polySia with a concentration of 1 g/L was used.

**Figure 2:**
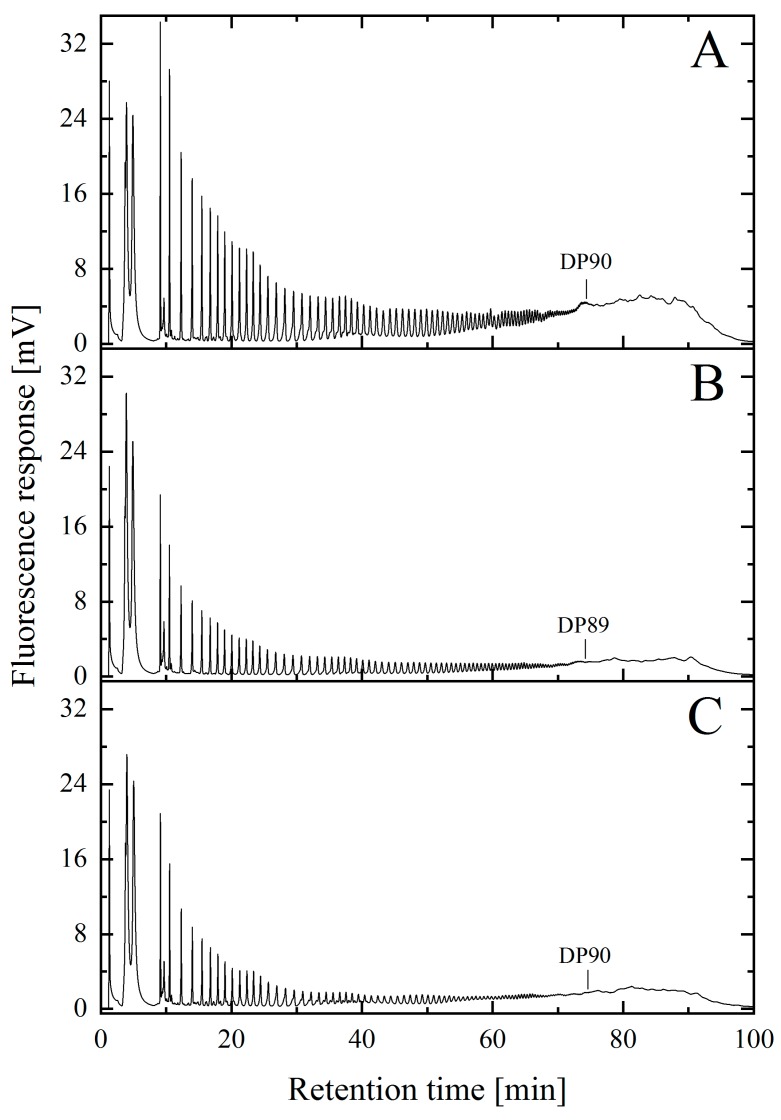
Characterization and comparison of the maximal polysialic acid chain length. PolySia with high endotoxin levels (**A**) was incubated at alkaline pH (pH > 13) (**B**) or purified via affinity columns (**C**) to reduce the endotoxin load. The degree of polymerization (DP) was measured using 1,2-diamino-4,5-methylenedioxybenzene (DMB)-HPLC analysis. The results show that the maximal chain length of the polySia was ≈DP90 and was not reduced following long-term incubation in an alkaline sodium hydroxide solution.

As shown in Figure 2, the maximum chain length of the initial polySia material was ≈DP90 (Figure 2A) and a comparable chain length was determined for NaOH-treated material (Figure 2B). The results obtained from the DMB-HPLC were also comparable to those published in earlier studies [22,23]. Thus, the alkaline treatment did not alter the length of the polymer chains. The control purified with EndoTrap HD columns under physiological conditions also showed no difference in the maximal chain length (Figure 2C). In addition, the fluorescence intensity of the individual peaks in the chromatogram was comparable for all measured samples.

### 2.3. Structural Analysis of Purified PolySia Using NMR Spectroscopy

**Figure 3:**
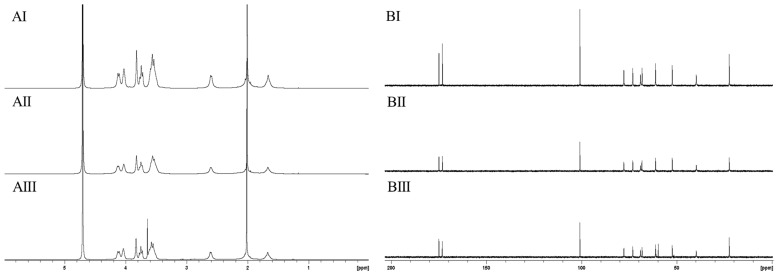
Comparison of the ^1^H- (**A**) and ^13^C- (**B**) NMR spectra of manufactured long-chain (LC) polySia with (**II**) and without (**I**) alkaline treatment for efficient removal of endotoxins. As a control, LC polySia was treated using a third approach with specific EndoTrap HD columns (**III**) under physiological conditions (pH = 7). The spectra of all three samples showed a high similarity. The NMR spectra of polySia purified using EndoTrap HD columns showed an additional signal at 60 ppm (^13^C) and 3.6 ppm (^1^H), indicating the presence of an unspecific impurity.

In recent decades, ^13^C-NMR spectroscopy has been applied as an efficient tool to determine the molecular structures of various polysaccharides [35]. In order to exclude possible hydrolysis effects or chemical modification, such as deacetylation, polySia was subjected to ^1^H- and ^13^C-NMR spectroscopy. The ^1^H- and ^13^C-spectra of manufactured NaOH-treated (Figure 3AII,BII) and untreated material (Figure 3AI,BI) were compared with spectra of untreated material. PolySia polished using EndoTrap HD columns was used as a control (Figure 3AIII,BIII).

All recorded ^13^C-NMR spectra (Figure 3B) showed the eleven characteristic signals for polySia. The two signals at a chemical shift of 173 ppm and 175 ppm represent the presence of two carbonyl groups in all spectra. Furthermore, all three spectra show a high similarity. However, in the ^13^C-NMR spectra of the control (Figure 3BIII), an additional signal appeared at a chemical shift of 60 ppm, indicating the presence of a small unspecific alcohol- or ether-containing impurity. The recorded NMR spectra indicated no presence of any chemical modification in the molecular monomer structure and are comparable with the spectra of α-(2,8)-polySia published in earlier studies [22,36].

The recorded ^1^H-NMR spectra shown in Figure 3A are also nearly identical and show a good correlation. The integration of all peaks amounted to twelve hydrogen atoms, which did not indicate any structural change in polySia caused by NaOH treatment or affinity chromatography. However, it should be taken into account that the H-atom bound to the amide function resonated at a chemical shift of 8 ppm. In order to give a better resolution in the figure, this range was not shown. The intense signal at a chemical shift of approximately 2 ppm indicates the presence of the methyl group at the N-acetyl residue. This signal was identified in all three spectra, indicating the presence of the characteristic N-acetyl residue. An additional signal with a chemical shift of 3.6 ppm was also identified in the ^1^H-NMR spectrum of LC polySia polished using EndoTrap HD columns (Figure 3AIII). This result confirms the identification of the slight impurity, which was also visible in the ^13^C-NMR spectrum.

### 2.4. Characterization of the Thermal Hydrolysis Conditions for Low-Molecular-Weight PolySia

**Figure 4:**
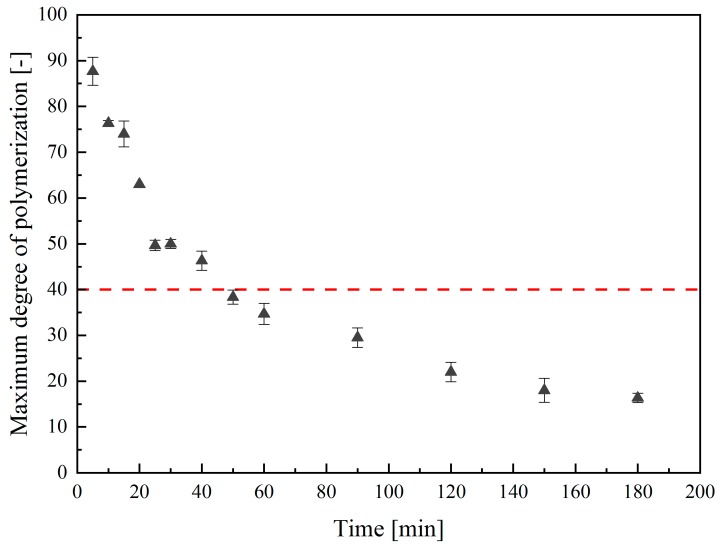
Maximum degree of polymerization as a function of hydrolysis time at 90 °C. LC polySia was subjected to a 3-h thermal hydrolysis at 90 °C. The measurement was carried out in triple determination and then the maximum degree of polymerization was subsequently determined using DMB-HPLC.

PolySia avDP20 can be efficiently produced from biotechnologically-manufactured LC polySia [17]. Since the glycosidic linkage of Neu5Ac is known to be acid-sensitive, mild hydrolysis enables the easy and cost-efficient production of polySia avDP20 [12,37]. Unfortunately, acidic conditions favor the internal lactonization between the carboxyl group and the adjacent 9-hydroxy group to a stable six-headed ring in oligoSia [30,31]. Stability studies showed that polySia decomposes in aqueous solution within a few days at room temperature. Earlier studies used this temperature sensitivity to produce low-molecular-weight polySia (polySia avDP20) by increasing the temperature [17,27]. However, it is also important to ensure that the working conditions do not cause any chemical changes in the molecular structure. To investigate this, thermal hydrolysis was conducted with biotechnologically produced LC polySia diluted to a concentration of 1 g/L in deionized water. PolySia was incubated at 90 °C and the process was monitored via DMB-HPLC. The development of the maximum chain length over time is displayed in Figure 4.

The results show that the LC polySia was effectively cleaved using incubation at 90 °C. The hyperbolic graph depicts an intense polymer chain shortening over time. After 10 min, the maximum chain length was already reduced to DP76 ± 1. As the process progressed, not only an increase in the proportion of short-chain polySia, but also a decrease in the maximum chain length was observed. After 40 min, the maximum chain length was determined at DP46 ± 2, which is almost half of the initial length. At the end of the 3-h treatment, the maximum DP was reduced from 90 ± 2 to 16 ± 1.

### 2.5. Isolation and Characterization of Low-Molecular-Weight PolySia avDP20

**Figure 5:**
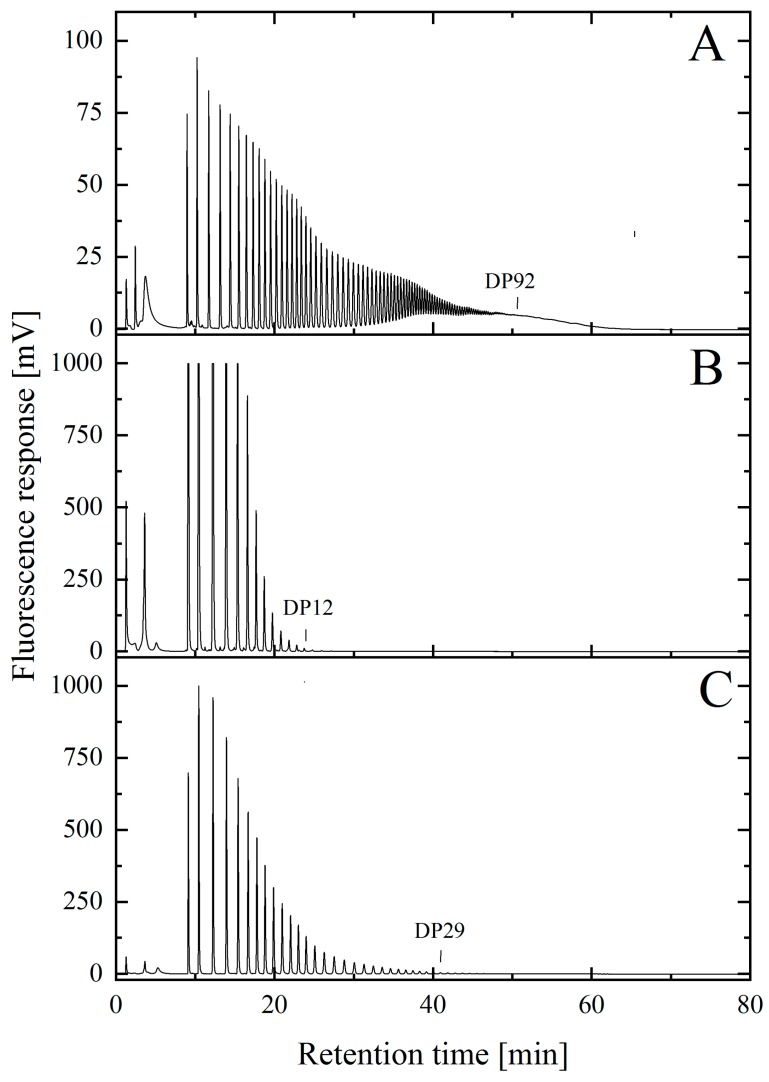
DMB-HPLC chromatograms of the isolated low-molecular-weight polySia after thermal hydrolysis. LC polySia (**A**) was incubated at 90 °C for 2 h. The hydrolyzed material was then collected via a three-stage gradient using an anion exchange (AEX) membrane adsorber. The first stage used 0.28 M ammonia bicarbonate for the elution of undesired short chain polySia molecules up to DP12 (**B**). The target fraction was eluted with 0.35 M ammonia bicarbonate and contained polySia with a chain length of DP29 (**C**).

After the working parameters for production of polySia avDP20 were investigated, thermal hydrolysis for production of low-molecular-weight polySia with a chain-length distribution of DP14–26 was performed. Therefore, LC polySia was dissolved in deionized water and hydrolyzed for 2 h at 90 °C. A three-stage gradient using AEX membrane adsorbers (SartobindQ nano 3 mL) was used for the targeted isolation of polySia avDP20. The first step was to elute the polySia, which was smaller than DP14. The second stage represented the target fraction and contained polySia < DP26. The third step was to elute the remaining material with a larger chain length. All fractions of the first two steps were combined and freeze-dried. The chain-length distribution of the target fraction was then determined using DMB-HPLC analysis (Figure 5).

The chromatograms show that the maximum chain length of the initial polySia material was ≈DP92 (Figure 2A). As expected, the maximum chain length of polySia was reduced using thermal hydrolysis. As shown in Figure 5B, polySia was eluted in the first gradient step with a maximum degree of polymerization of DP12. The concentration of ammonium bicarbonate was 0.28 M. In the second step, the concentration of ammonium bicarbonate was increased to 0.37 M, allowing for a maximum chain length of DP29 to be isolated. The target fraction contained polySia in a chain length range of DP20 ± 8.

### 2.6. Analysis of the Intramolecular Stability of Thermal Hydrolyzed Low-Molecular-Weight PolySia

**Figure 6:**
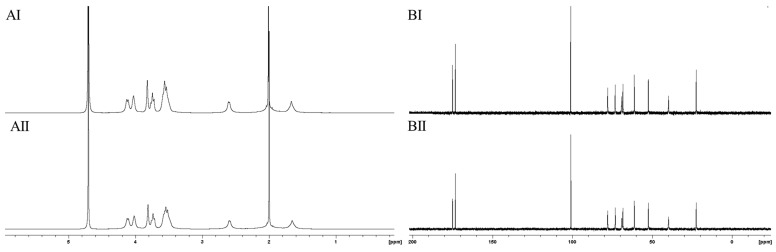
Comparison of the ^1^H- (**A**) and ^13^C- (**B**) NMR spectra of LC polySia (**I**) and hydrolyzed polySia (**II**). Hydrolysis was performed for 3 h at 90 °C and polySia was subsequently collected with an AEX membrane adsorber (SartobindQ 75 mL). The spectra show no changes of the molecular structure.

Earlier studies revealed that α-glycosidically-linked Neu5Ac shows potential instability toward acidic conditions and that temperature plays a key role in affecting the hydrolytic reactions of polySia [27]. Therefore, the working conditions for thermal hydrolysis can pose the risk of inducing intra-molecular degradation reactions in the material [38]. To obtain an adequate quantification of potential cleaving effects on short-chain polySia caused by thermal hydrolysis, ^1^H- and ^13^C-NMR spectroscopy was applied to analyze the structural integrity of the low-molecular-weight polySia. The ^1^H- and ^13^C-NMR spectra of initial LC polySia (Figure 6AI,BI) were compared with the spectra of hydrolyzed material with a low molecular weight (Figure 6AII,BII).

Both recorded ^13^C-NMR spectra showed the eleven characteristic absorption signals of the C-atoms of polySia. The spectra have a high similarity and are even comparable with the recorded spectra of LC polySia during downstream processing (Figure 3B). The two characteristic signals indicating the presence of both carbonyl groups were visible at a chemical shift of 173 ppm and 175 ppm. The corresponding ^1^H-NMR spectra (Figure 5A) were similar to the recorded ^1^H-NMR spectra (Figure 3A) after downstream processing and endotoxin removal. The recorded NMR spectra indicated that thermal hydrolysis had no structural influence on the monomer structure of the low-molecular-weight polySia. In addition, the NMR spectra of LC polySia (Figure 3) and low-molecular-weight polySia can be compared with already published results [22,36].

## 3. Discussion

In this study, the intramolecular structure of biotechnologically-produced LC polySia and low-molecular-weight polySia was analyzed and investigated to determine whether chemical modifications were induced by the hydrolyzing effects of temperature or alkaline treatments. First, polySia was biotechnologically produced in *E. coli* K1 using a production process that has been described elsewhere [15]. After downstream processing, the material was purified to remove proteinogenic impurities and DNA while endotoxin levels remained above 3000 EU per mg of the final product. The observed endotoxin burden was similar to that reported in earlier studies [15]. This process was performed in order to maintain endogenous polySia material with an intact monomer structure.

To further reduce the endotoxins, the material was incubated in alkaline sodium hydroxide solution and subsequently collected using an AEX SartobindQ 75 mL membrane adsorber. Measurement of the endotoxin burden revealed a reduction to below 5 EU per mg. The same initial material was polished under physiological conditions through the application of GMP-compliant affinity columns for the specific removal of endotoxins. The application of EndoTrap HD columns resulted in a final level of 2 EU per mg. Both techniques were therefore suitable for the production of pharmaceutical grade polySia under GMP conditions [18,39]. However, EndoTrap HD columns are very expensive, can accommodate only small volumes per columns (1 mL), and use a gravitational flow, restricting their use to polishing only small amounts of polySia. Upscaling to a large-scale application would significantly increase the costs of production. In contrast, a NaOH treatment enables the purification of polySia concentrations, even at an industrial scale [16], and can be easily adapted to varying batch sizes.

Subsequently, the freeze-dried product was analyzed in terms of potential cleavage or chemical modification effects due to the harsh conditions of the sodium hydroxide treatment. EndoTrap HD columns can be used under physiological pH-conditions and therefore served as a control. First, the maximum chain length was analyzed using DMB-HPLC [40]. Due to the partial decay, it was only possible to accurately detect monomers up to long-chain polySia with a degree of polymerization of 90 using this method. The chain length of polySia was determined by counting the number of individual peaks. A comparison of the chain lengths achieved with the different approaches revealed no significant differences in the affinity matrices or in the alkaline incubation. The maximum chain length was determined to be approximately DP90 for all samples. However, derivatization was conducted under acidic conditions, which may have resulted in the partial hydrolysis of the polySia. A quantitative determination of the concentration of individual degrees of polymerization was not possible [23].

Thermal hydrolysis was performed to enable the efficient production of pharmaceutically relevant short-chain polySia. In contrast to acid-catalyzed hydrolysis and enzymatic degradation [27,41], thermal hydrolysis requires significantly less resources since the subsequent removal of chemicals or enzymes is not necessary [17]. From an economic point of view, thermal hydrolysis is also the most cost-effective approach. Enhancement of the temperature to 90 °C accelerates the decay of the polymer, whereas the maximum chain length was halved within one hour. Unfortunately, a targeted reduction of the chain-length distribution of the product is rather complicated since the chain-length distribution of the fission products can only be influenced by varying the hydrolysis time and duration. Nevertheless, the method holds significant potential for the cost-effective production of low-molecular-weight poly- and oligoSia without causing chemical modifications, as demonstrated in the ^1^H- and ^13^C-NMR studies.

## 4. Materials and Methods

### 4.1. Chemicals and Buffer

Bulk chemicals were purchased from Sigma-Aldrich (Taufkirchen, Germany) or Carl Roth GmbH & Co.KG (Karlsruhe, Germany). Deionized water was prepared with ARIUM^®^ (Sartorius Stedim Biotech, Göttingen, Germany).

### 4.2. Bacterial Strains and Strain Maintenance

The producing strain used in this study was *Escherichia coli* B2032/82 serotype K1 (DSM-Nr. 107,164), an original clinical isolate [42,43]. Glycerol stocks were prepared as previously reported [15]

### 4.3. Production of PolySia in E. coli K1

The production of long-chain polySia in *E. coli* K1 was carried out as previously reported using a chemically defined medium that supported the optimal synthesis of polySia [15,44]. Following downstream processing, endogenous polySia with a maximal chain length of >DP90 and an endotoxin burden above 3000 EU per mg polySia was produced.

### 4.4. Endotoxin Removal Using Combinatorial NaOH Treatment and AEX Membrane Adsorbers

Long-chain material was treated with NaOH for a further reduction of the endotoxin burden. PolySia was dissolved in 100 mM sodium phosphate buffer in a maximum concentration of 2 g/L. The subsequent steps were conducted as described previously [16]. Afterward, the product was collected using SartobindQ^®^ 75 mL (membrane area: 2700 cm^2^, column volume (CV): 75 mL) (Sartorius Stedim Biotech, Göttingen, Germany). The membrane adsorber was cleaned and flushed according to the manufacturer’s instructions. For equilibration, 5 CV of 0.1 M ammonium bicarbonate, pH 7.4 (running buffer), was used. Afterward, the pretreated sample was loaded onto the membrane adsorber. Non-bound material was removed via a washing step with 5 CV running, and finally, the sample was collected via the isocratic elution of 5 CV 0.75 M NaCl. During all steps, a flow rate of 100 mL/min was used. Dialysis and freeze drying were performed as previously reported [16].

### 4.5. Endotoxin Removal Using EndoTrap^®^ HD Columns

Affinity columns with specific bacteriophage ligands to reduce the endotoxin load of the produced polySia were operated under physiological conditions, whereby structure impairment was avoided. The hydrophilic affinity matrix column (EndoTrap^®^ HD 5/1, Hyglos GmbH, Bernried, Germany) was equilibrated with 6 CV of 50 mM potassium phosphate buffer (pH 7) containing 0.7 M NaCl and 1 mM CaCl_2_. Subsequently, a 5 g/L solution of endogenous polySia with >3000 EU/mg dissolved in equilibration buffer was applied as a sample to the column and collected as a flow-through. For regeneration, 3 CV of manufacturer’s regeneration buffer (20 mM (4-(2-hydroxyethyl)-1-piperazineethanesulfonic acid (HEPES), 1 M NaCl, 2 mM Ethylenediaminetetraacectic acid (EDTA), pH 7.5) was applied to the column.

### 4.6. Thermal Hydrolysis for the Production of Low-Molecular-Weight PolySia

For the production of low molecular weight polySia, the endogenous long-chain material was dissolved in deionized water in a concentration of 1 mg/mL and aliquoted as a triplet with a sample volume of 1 mL. The samples were then incubated at 90 °C and 500 rpm in a heating block. Samples were taken continuously over a period of 3 h, and the maximum chain length was determined.

### 4.7. Fractionation of Short Chain PolySia

The isolation of short chain polySia was implemented using an AEX membrane adsorber (SartobindQ^®^) in a fast protein liquid chromatography (FPLC) system as described previously [17].

### 4.8. Quantification of PolySia Concentrations

For the quantification of the polySia concentration, a colometrically modified TBA assay was conducted as described previously [15]. For calibration, commercially available colominic acid (Carbosynth, Compton, United Kingdom) was used in concentrations ranging from 0.05 to 1 g/L and deionized water as a blank.

### 4.9. DMB-HPLC Analytics for Chain Length Characterization of PolySia

Possible impacts on the maximum chain length of the product due to long-term incubation under alkaline conditions (pH > 13) were analyzed using common 1,2-diamino-4,5-methylenedioxybenzene (DMB) HPLC analysis [40]. Sample preparation through DMB-derivatization and HPLC analysis was conducted as described previously [17].

### 4.10. Structure Analysis Using ^1^H- and ^13^C-NMR Spectroscopy

^1^H- and ^13^C-nuclear magnetic resonance (NMR) spectra were recorded on an Ascend 400 MHz with Avance III console and Prodigy BBFO probe (Bruker BioSpin GmbH, Rheinstetten, Germany). Spectra were recorded at 25 °C. The solid samples were dissolved in D_2_O to a maximum concentration of 30 mg/mL. The spectra were referenced with respect to the residual solvent signal of HDO.

### 4.11. Determination of Endotoxins

The endotoxin concentration was determined using the Endosafe^®^ nexgen-PTS™ system (Endosafe^®^ nexgen-PTS™, Charles River Laboratories, Boston, MA, USA).

## 5. Conclusions

Low-molecular-weight polySia with a polymerization degree of 20 holds considerable potential for therapeutic applications to treat neurodegenerative diseases. Large-scale production can be implemented through the cultivation of *E. coli* K1 with yields of an acceptable range. Via incubation in alkaline solutions and recovery using AEX membrane adsorbers, the endotoxin burden in the final product was cheaply and easily reduced to levels down to 2 EU per mg, which is in the range for medical applications. The method can also be upscaled to meet large-scale requirements and polymer chain lengths were not affected by NaOH. Synthesis of pharmaceutically relevant polySia avDP20 was achieved through thermal hydrolysis. Although the method is probably the cheapest and easiest way to produce low-molecular-weight polySia, a specific synthesis of molecules with defined chain lengths was only possible to a limited extent. Due to this unspecificity, only the range of the chain-length distribution could be limited by varying the reaction time. Furthermore, the NMR studies showed that neither alkaline treatment nor thermal hydrolysis caused intramolecular changes in the monomer structure. In addition, no chemical deacetylation of the molecule was observed. Thus, this study proved that a combinatorial application of the described method allowed for the production of high-purity, low-molecular-weight polySia without negatively affecting the structure.
